# Critical incident reporting system: Is it the solution?

**DOI:** 10.4103/1658-354X.71131

**Published:** 2010

**Authors:** Abdelazeem El-Dawlatly

**Affiliations:** *Professor of Anaesthesia, College of Medicine, King Saud University, Riyadh, Kingdom of Saudi Arabia. E-mail: dawlatly@ksu.edu.sa*

Critical incident report (CIR) is defined as a report of any untoward and preventable mishap which is associated with the administration of general or regional anesthesia and which leads to, or could have led to, an undesirable patient outcome. So far, we have published three reports of the critical incidents which were reported and discussed in our monthly morbidity and mortality meeting. Our first report covers the period from 1991 to 1997, during which 143 incidents were reported to the department. Etiologies of critical incidents were organization rules, technique, patient environment, human factor, team work and lack of communication.[1] Our second report covers the period from 1998 to 2002, during which 71 incidents were reported to our department. The following reasons were given: human factor, team communication, patient condition and technical problems.[2] Our third report covers the period from 2003 to 2008, in which 70 incidents were discussed. Incidents were classified depending on the cause as follows: pulmonary, cardiovascular, central nervous system, metabolic, inadvertent drug injection, communication failure, equipment failure and miscellaneous causes. Most of the incidents happened during maintenance of anesthesia, followed by during induction then at the same operative day. Respiratory events reported for the majority of cases followed by communication failure[3]. Recently Merry *et al*, have published two articles on international standards of patient safety and its implementation[4,5]. It seems that there is now major concerns among anesthesia community on implementing the standard of care in an attempt to improve patient safety in anesthesia practice. As one might have noticed, the reasons leading to the incidents repeat themselves in these reports, but less frequently. In this issue of *Saudi Journal of Anaesthesia*, Al Saeed has stated that the lowest number of accused claims which were presented to the legal health organization in Saudi Arabia was from university hospitals.[4] This will draw our attention to the importance of CIR system in improving patient safety and will force all health sectors to support CIR systems and education programs as university hospitals do. Obviously, there are some limitations of implementing CIR system in health care services other than university vicinity. Though the CIR system may improve the patient outcome, it will bring the incidents on surface with the fear of subsequent legal action. On the contrary, it will improve learning objectives in anesthesia practice and, subsequently, will prevent repeating the same incidents.
